# Glycosaminoglycan-Inspired Biomaterials for the Development of Bioactive Hydrogel Networks

**DOI:** 10.3390/molecules25040978

**Published:** 2020-02-21

**Authors:** Mariana I. Neves, Marco Araújo, Lorenzo Moroni, Ricardo M.P. da Silva, Cristina C. Barrias

**Affiliations:** 1 i3S-Instituto de Investigação e Inovação em Saúde, Universidade do Porto, Rua Alfredo Allen 208, 4200-135 Porto, Portugal; mariana.neves@i3s.up.pt (M.I.N.); marco.araujo@i3s.up.pt (M.A.); 2 INEB-Instituto de Engenharia Biomédica, Universidade do Porto, Rua Alfredo Allen 208, 4200-135 Porto, Portugal; 3 FEUP-Faculdade de Engenharia da Universidade do Porto, Departamento de Engenharia Metalúrgica e de Materiais, Rua Dr Roberto Frias s/n, 4200-465 Porto, Portugal; 4 MERLN Institute for Technology-Inspired Regenerative Medicine, Maastricht University, 6229 ET Maastricht, The Netherlands; l.moroni@maastrichtuniversity.nl; 5 ICBAS-Instituto de Ciências Biomédicas Abel Salazar, Universidade do Porto, Rua de Jorge Viterbo Ferreira 228, 4050-313 Porto, Portugal

**Keywords:** GAG, hydrogels, hybrid systems, biomaterials, polysaccharides, proteins, self-assembly peptides, GAG-mimetics

## Abstract

Glycosaminoglycans (GAG) are long, linear polysaccharides that display a wide range of relevant biological roles. Particularly, in the extracellular matrix (ECM) GAG specifically interact with other biological molecules, such as growth factors, protecting them from proteolysis or inhibiting factors. Additionally, ECM GAG are partially responsible for the mechanical stability of tissues due to their capacity to retain high amounts of water, enabling hydration of the ECM and rendering it resistant to compressive forces. In this review, the use of GAG for developing hydrogel networks with improved biological activity and/or mechanical properties is discussed. Greater focus is given to strategies involving the production of hydrogels that are composed of GAG alone or in combination with other materials. Additionally, approaches used to introduce GAG-inspired features in biomaterials of different sources will also be presented.

## 1. Introduction

**Figure 1:**
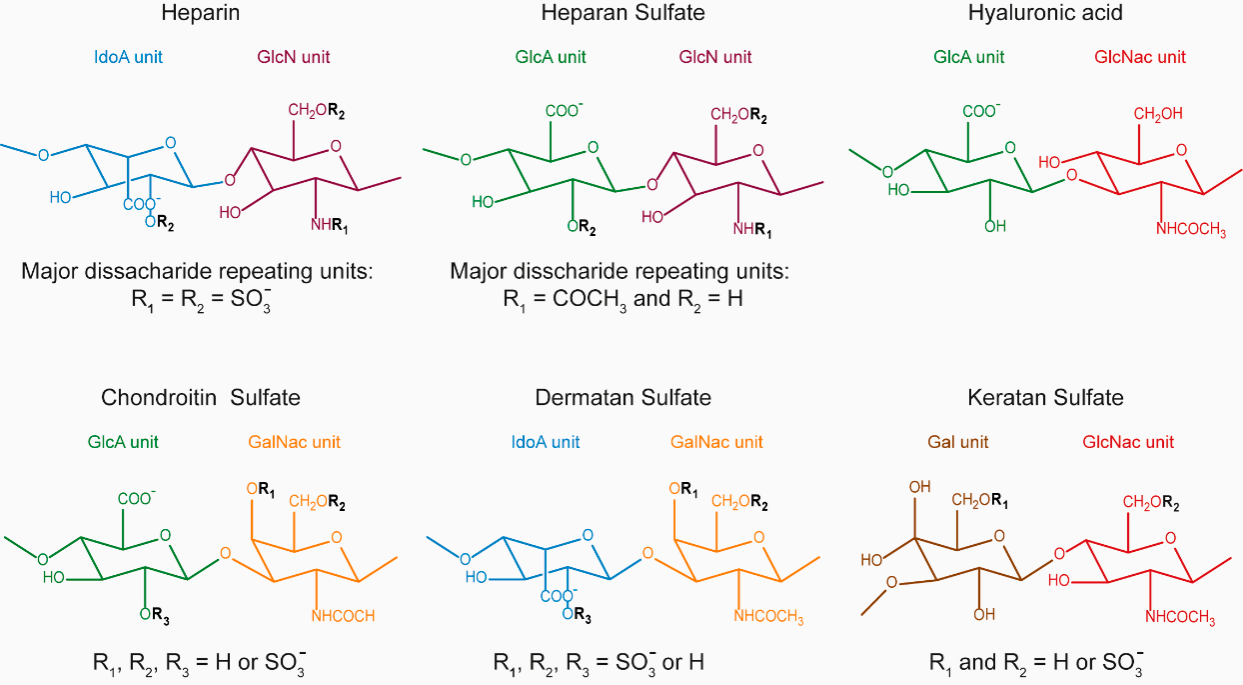
Structure of Glycosaminoglycans (GAG) molecules according to main disaccharide composition. GAG are composed of combined uronic acids [β-d-glucuronic acid (GlcA) or α-l-iduronic acid (IdoA)] and amino sugars [α-d- or β-d-glucosamine (GlcN), N-acetylglucosamine (GlcNac) or *N*-acetyl-galactosamine GalNac)]. From the combination of the different uronic acid and amino sugars, four main groups of GAG can be distinguished. (1) Heparin and Heparan Sulfate, R_1_=COCH_3_ or SO_3_^−^ and R_2_=H or SO_3_^−^; Heparan Sulfate, R_1_=COCH_3_ or SO_3_^−^ and R_2_=H or SO_3_^−^. Heparin and Heparan Sulfate share a common amino sugar (α-D-GlcN) but differ in their major uronic acid unit (α-l-IdoA in heparin and β-d-GlcA in heparan sulfate), linked by 1-4 glycosidic bonds. (2) Chondroitin Sulfate and Dermatan Sulfate also share the same amino sugar unit (GalNac) but their uronic unit is β-d-GlcA and α-l-IdoA, respectively. Chondroitin sulfates can be classified according to the position of the sulfates: R_1_=R_2_=R_3_=H (nonsulfated chondroitin), R_1_=SO_3_^−^ and R_2_=R_3_=H (chondroitin-4-sulfate, CSA), R_1_=R_3_=SO_3_^−^ and R_2_=H (chondroitin-2,4-disulfate, CSB), R_2_=SO_3_^−^ and R_1_=R_3_=H (chondroitin-6-sulfate, CSC), R_2_=R_3_=SO_3_^−^ and R_1_=H (chondroitin-2,6-disulfate, CSD), R_1_=R_2_=SO_3_^−^ and R_3_=H (chondroitin-4,6-disulfate, CSE), R_1_=R_2_=R_3_=SO_3_^−^ (trisulfated chondroitin). CS and DS can be found as co-polymeric structures and despite the difference in the uronic unit, DS is also known as chondroitin sulfate B (CSB). (3) Hyaluronan, or hyaluronic acid, is composed of β-1,4-d-GlcA and β-1,3-*N*-acetyl-d-GlcNac repeating units and is the only non-sulfated GAG. (4) Keratan sulfate has the particularity of not containing uronic acids, being composed of alternating units of galactose (β-1,3-d-Gal) and β-1,4-N-acetyl-d-GlcNac.

Glycosaminoglycans (GAG) are a family of glycans comprising long, linear polysaccharides that display a wide range of relevant biological roles. GAG share common repeating, alternating disaccharide units, composed of combined sulfated or non-sulfated uronic acids (glucuronic acid or iduronic acid) and amino sugars (glucosamine or galactosamine) [1]. From the combination of different uronic acid and amino sugars, four main groups of GAG can be distinguished: heparin (HEP) and heparan sulfates (HS), chondroitin sulfate (CS) and dermatan sulfate (DS), keratan sulfate (KS) and hyaluronic acid (HA, also known as hyaluronan) (Figure 1). GAG can occur with different sulfation degrees and patterns that affect their biological function [2], with exception of HA, the only non-sulfated GAG. Examples of biofunctional modulation include the increased anticoagulant activity of oversulfated CS [3] or distinct regulation of signaling pathways and cellular response according to GAG sulfation degree [4,5]. Differences in GAG chain elongation or molecular weight can also affect their biological action. For instance, high molecular weight HA is reported to have an anti-inflammatory role, whereas low molecular weight HA is reported to induce pro-inflammatory responses [6,7,8,9].

**Figure 2:**
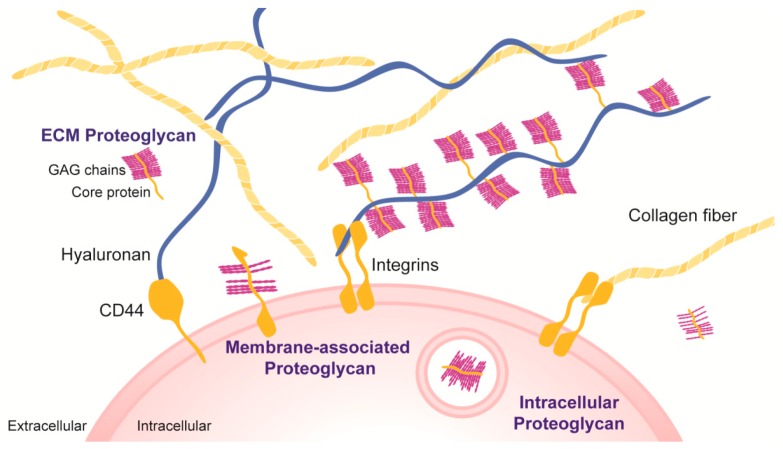
Localization of GAG and PG in native tissues. GAG associate covalently to core proteins forming proteoglycans (PG), with exception of HA. Instead, HA physically interacts with other GAG and PG through specific binding domains, as well as particular cell receptors (e.g., CD44). PG can be classified according to their location: They can be found inside cells in secretory compartments where they support storage of positively charged biomolecules (intracellular PG); at the cell membrane as membrane-associated PG where they are involved in cell–cell and cell–ECM interactions (membrane-associated PG) or be secreted to the ECM, where they play both biological and structural roles (extracellular PG).

Most GAG are covalently attached to core proteins, forming proteoglycans (PG). HA is one exception, occurring as a free GAG, able to physically interact with other GAG and PG through specific binding domains. HEP also presents a distinctive feature, as it is typically released from mast cells attached to a small peptide, following cleavage from its core protein [10]. GAG and PG can be located either inside cells (secretory compartments), at the cell membrane (membrane-associated PG) or in the extracellular matrix (ECM), upon secretion (Figure 2).

Herein, a major focus will be given to ECM GAG and their applications in the development of new biomaterials for Tissue Engineering (TE) and Regenerative Medicine (RM). This is motivated by the implication of ECM GAG and their PG in a wide variety of biological processes, including embryonic development [11,12], infection [13], inflammation [6,14], wound healing [8] and cancer [14,15,16]. Variation in ECM GAG content and composition is also tissue-specific, age-dependent [17,18] and known to be associated with certain pathologies [19,20].

The ECM is an extremely dynamic environment, affording physical protection against mechanical stress, allowing in and out biomolecules diffusion and providing both biochemical and biomechanical signaling cues to cells. In the ECM, one major role of GAG relies on their ability to interact with other biological molecules, including matrix proteins, growth factors, chemokines and proteases. Some of these interactions present high affinity and selectivity [21]. Such interactions are believed to occur not only via electrostatic interactions, due to the high negatively charged nature of GAG, but also via ionic interactions and hydrogen bonding [10], being dependent on GAG composition and structure [22]. This allows ECM GAG to act as “storage” components, protecting biological molecules from proteolysis [23] or inhibiting factors [24], while allowing the generation of chemotactic gradients within the ECM. Another pivotal role of ECM GAG is their ability to confer mechanical stability to tissues. Due to their charged nature, GAG are highly hydrophilic macromolecules. Their capacity to retain high amounts of water promotes ECM hydration, conferring resistance to compressive forces, particularly in high load-bearing tissues, such as cartilage, allowing desorption and resorption processes. Additionally, mechanical stability is improved by GAG/PG interaction with several proteins present in the ECM, particularly collagen [25,26].

Minding the biological and structural roles provided by GAG in the ECM, they stand out as useful tools in the design of biomimetic networks, either alone or in combination with other materials. In biomedical applications, particularly TE and RM, biomaterials have been developed for creating microenvironments that partially recapitulate the composition and properties of the ECM of native tissues. In the past, bioinert materials were often chosen for developing strategies for tissue regeneration, as they presumably avoided undesirable host responses. In more recent years, however, the paradigm shifted towards the use of biointeractive, dynamic systems able to either induce particular cellular responses [27,28,29] (i.e., cell instructive) and/or be themselves modulated by cells [30,31] (i.e., cell responsive) or the surrounding environment [32,33,34,35,36].

In this context, this review focuses on the use of GAG for producing 3D networks with improved biological activity and mechanical properties. Particular focus will be given to hydrogels, highly hydrated networks that intrinsically recapitulate some structural features of the native ECM. The first section will explore strategies used in the development of hydrogels composed only of GAG. Subsequently, a section devoted to hydrogel hybrid networks will explore the development of systems combining GAG with other materials, particularly peptides and proteins, non-mammalian polysaccharides and decellularized ECM. The rational and the impact of including GAG in those systems will be discussed in detail. Finally, strategies for incorporating GAG-inspired features in different types of materials, so-called GAG-mimetics, will be described and explored in the last section.

## 2. GAG-Based Hydrogels

Hydrogels composed only of GAG are mainly based on CS or HA derivatives. This is most likely related to their key biological and structural functions in the ECM but also because they are major components of tissues like cartilage or skin, respectively. The prevalence of CS and HA in these tissues occurs in several mammalian and non-mammalian species, which may also facilitate the extraction and purification processes of these GAG.

Different types of GAG-based biomaterials have been described and characterized as potential candidates for biomedical applications, particularly for cartilage [37,38] and neural [39,40] tissues. While non-modified GAG can be used [41,42], most strategies resort to chemical modifications to add specific moieties to GAG. Such functionalizations allow the incorporation of new features to modulate properties such as biological performance, compound delivery profile, mechanical properties and degradation pattern.

In this section, strategies used to develop GAG-based hydrogels will be categorized as approaches intended for modulation of hydrogel network formation or modulation of scaffold (bio)degradation.

### 2.1. Modulating Hydrogel Network Formation

Commonly reported GAG modifications are motivated by the need for rendering GAG crosslinkable and modulate network formation to produce hydrogels with enough structural stability. Besides, these systems provide opportunities for developing materials that may present partially different chemical composition without critically compromising their structure. This allows, for instance, the comparison of hydrogels composed of the same GAG backbone but with varying sulfation degree or pattern without critically compromising the formation of a network. Frequently, strategies for creating GAG-based hydrogels rely on irreversible covalent bonding between GAG chains, but reversible crosslinking strategies are being further explored to develop GAG-based dynamic environments, as it will be explored on the following subsections.

#### 2.1.1. Light and Temperature Induced Crosslinking

**Figure 3:**
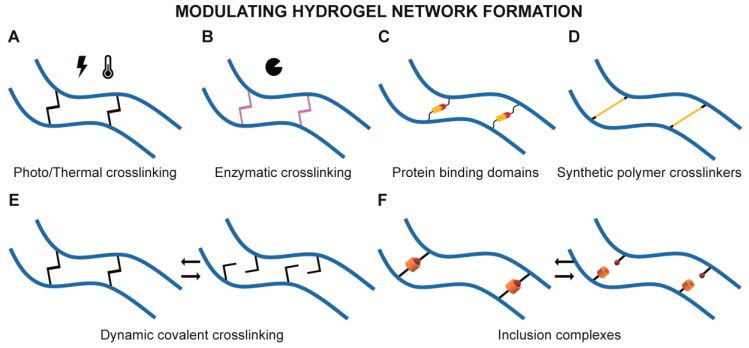
Schematic representation of main strategies to modulate network formation of GAG-based hydrogels. GAG-hydrogels can be produced from chemical or physical crosslinking between GAG chains, requiring the production of GAG-derivatives with new functionalities. Such modifications can be performed to include new functional groups [e.g., (meth)acrylates, thiols, tyramines, coumarins, peptide sequences] able to crosslink when exposed to light/temperature (**A**) or enzymes (**B**). Incorporation of protein-binding domains (**C**) with high selectivity towards one another can also be used as strategy to covalently crosslink GAG. Combination of GAG with smaller synthetic polymer crosslinkers (**D**) can be achieved for non-modified GAG or their derivatives. Additionally, GAG-based hydrogels can be designed to have reversible crosslinking, obtained by covalent dynamic crosslinking (e.g., hydrazone bonds or coumarin photo-induced reversible cyclodimerization) (**E**), or by producing physical hydrogels with GAG derivatives able to crosslink via inclusion complexes (e.g., host-guest cyclodextrin complexes) (**F**).

Photo or thermal-induced covalent crosslinking (Figure 3A) is one of the most used strategy to create covalent hydrogels. A typical approach is the incorporation of photosensitive chemical groups [37,38,43] in GAG that allow their covalent crosslinking upon exposure to light and in the presence of a photoinitiator. In fact, covalent crosslinking systems allow the production of hydrogels exclusively composed of GAG or their derivatives with a fine tuning of their final structural composition. For instance, photocrosslinking strategies allow a great control over the crosslinking process, both spatially and temporally, by adjusting light wavelength, intensity and exposure time. GAG crosslinking density can also be modulated by synthesizing derivatives with different modification degrees (i.e., substitution degrees) that impact the final properties of produced hydrogels. For instance, Ornell KJ et al. [44] reduced the swelling ratio of CS hydrogels by increasing the amount of photosensitive groups in the GAG backbone.

**Figure 4:**
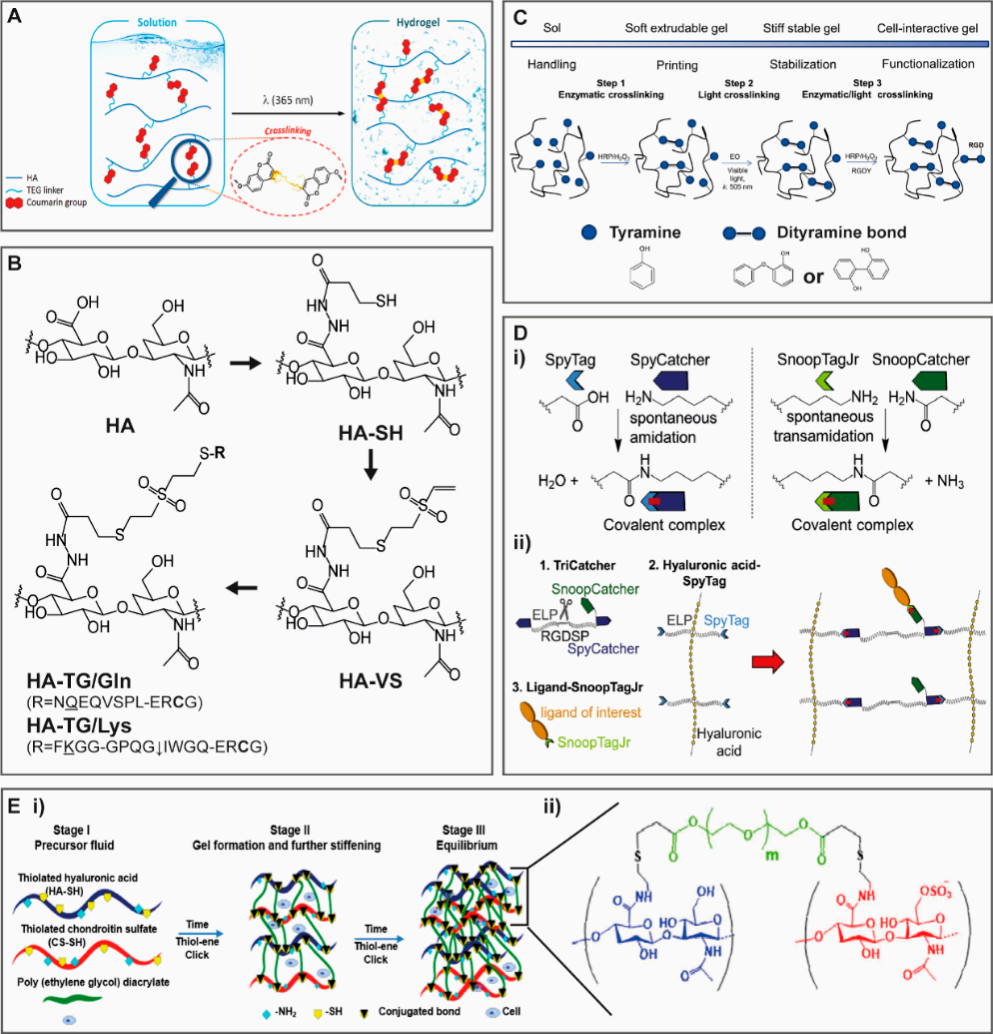
(**A**) Representation of coumarin derivatized HA undergoing crosslinking when exposed to near-UV light stimulus (λ = 365 nm) via photocycloaddition reaction between coumarin moieties and without any catalysts or radical initiators [45]. (**B**) Synthesis of HA derivatives incorporating transglutaminase (TG) peptides; thiol groups were incorporated in HA backbone via carbodiimide reaction (HA-SH); thiol groups were then substituted with vinyl sulfones (HA-VS) and subsequently with TG/glutamine (HA-TG/Gln) and TG/lysine (HA-TG-Lys) peptides via Michael addition; MMP cleavage site marked by an arrow, cysteines (C) that provide thiols for conjugation onto HA-VS are in bold and the Lys (K) and Gln (Q) covalently coupled to each other on their side chains by FXIIIa are underlined [39]. (**C**) Crosslinking steps of a HA-tyramine derivative bioink throughout the bioprinting process: step 1, the bioink is partially enzymatically crosslinked in the presence of HRP and H_2_O_2_ to improve extrusion; step 2, during extrusion, the bioink is exposed to green light to trigger photocrosslinking and stabilize the construct; step3, post-bioprinting, enzymatically driven functionalization of the scaffold to incorporate cell adhesive RGD motives [53]. (**D**) (i) SpyTag/SpyCatcher and SnoopTagJr/SnoopCatcher spontaneous amide bond formation; (ii) hydrogel network formation and functionalization with ligand of interest. Central sequence in TriCatchers may incorporate RGDSP-containing or MMP-cleavable linkers [56]. (**E**) (i) Representation of the HA/CS/PEGDA hydrogel and gelation process that leads to a polymeric network covalently crosslinked via thiol-ene Michael addition click reactions; (ii) chemical crosslinking of GAG thiol derivative mixtures, HA-SH (blue) and CS-SH (red), with PEGDA (green) by conjugate addition [58] (Reproduced with permission from [39,45,53,56,58]).

Other light-induced covalent crosslinking strategies have been explored that do not involve free radical polymerization, thus avoiding the need for initiator molecules. A GAG system based on the incorporation of coumarin groups in HA backbone was shown to promote covalent crosslinking by coumarin cyclodimerization, without the need for a radical initiator [45]. In this study, a coumarin group was linked to HA backbone through a triethylene glycol (TEG) side arm, and covalent crosslinking occurred between two coumarin groups, upon exposure to near-UV light (λ = 365 nm) (Figure 4A) [45]. Interestingly, while coumarin works as the covalent linker in the HA hydrogel, this compound and its derivatives are also reported to have, per se, relevant biological roles as anti-inflammatory [46,47], antioxidant [48] and anti-cancer [49] agents. This highlights the relevance of designing chemical modifications to incorporate moieties with a dual role, as these may contribute to structural stabilization, while also displaying bioactivity.

On the other hand, temperature-induced crosslinking allows the development of materials able to undergo gelation at relevant clinical temperature conditions, namely, body temperature (37 °C). As referred above, covalent systems can be used to study the effects of chemically distinct GAG. This can occur for hydrogels exclusively based on GAG, but even in cases where their final application is prospected for a multicomponent system. For instance, by modifying unsulfated chondroitin and CS with methacrylate groups, Lim JJ et al. [50] were able to study the effect of sulfation degree on CS interactions with positively charged growth factors. The authors were able to produce GAG hydrogels with different sulfation degrees by mixing unsulfated chondroitin and CS in varying mass ratios and inducing covalent crosslinking at 37 °C [50]. The authors observed a correlation between sulfation degree and retention of a positively charged model protein (histone) from CS hydrogels [50]. Unsulfated chondroitin and CS were then incorporated in poly(ethylene glycol) (PEG)-based hydrogels to study the effect of sulfation in mesenchymal stem/stromal cells (MSC) response [50]. As expected, PEG-based hydrogels with higher sulfation degree retained more transforming growth factor (TGF)-β1 [50]. Additionally, it was observed an increased responsiveness of encapsulated MSC to soluble chondrogenic cues in hydrogels containing desulfated chondroitin as opposed to the ones containing CS [50]. Such results indicate that interactions between chondroitins with different sulfation degree and biomolecules are not only distinctive but also ultimately affect their biological activity.

#### 2.1.2. Enzymatically Driven Crosslinking

GAG covalent crosslinking can also be induced enzymatically (Figure 3B) and advantageous in scenarios where milder processes are required while still enabling successful hydrogel production. For neural applications, Broguiere et al. [39] produced a HA-based scaffold where HA was modified with transglutaminase substrate peptide sequences, with either a glutamine or lysine reactive residues (Figure 4B). The authors were able to produce an injectable material with gelling time frames ranging from seconds to hours, according to the amount of enzymatic trigger added, the blood coagulation factor XIII (FXIIIa) [39].

Another reported approach of enzymatically-induced crosslinking consists on the conjugation of GAG with tyramine groups, via reactions catalyzed by horseradish peroxidase (HRP) and hydrogen peroxide (H_2_O_2_) [51]. HA-tyramine derivatives were used to produce printable bioinks for biofabrication strategies [52,53]. In the particular case of biofabrication applications, apart from the mechanical properties of the final product, the rheological properties of polymer solutions are also critical and should assure high bioprinting fidelity rates. In Petta et al. [53], an HA-tyramine derivative enabled covalent binding processes throughout different phases of the extrusion bioprinting processes with distinct finalities (Figure 4C): (i) prior to extrusion, it allowed enzymatic crosslinking to improve bioink printability; (ii) during bioprinting, secondary crosslinking could be induced upon visible light exposure using eosin Y as a photocatalyst; and finally, (iii) it allowed enzymatically-induced functionalization with cell adhesive peptides after bioprinting [53]. This strategy shows how a single chemical modification provides a versatile biomaterial that can undergo multiple crosslinking strategies, throughout different stages of scaffold production, improving both mechanical and biological features.

#### 2.1.3. Crosslinking with Protein Binding Domains

Covalently crosslinked GAG hydrogels were already produced from GAG modification with specific peptide sequences present in protein adhesive domains (Figure 3C). Howarth’s group [54] sliced the binding domain of a bacterial fibronectin-binding protein (FbaB) to form a peptide (SpyTag) and its protein partner (SpyCatcher). SpyTag and SpyCatcher covalently interact, spontaneously and irreversibly, via amide bond formation between lysine and aspartic acid residues, under a wide range of pH and temperature (Figure 4D(i), left) [54]. Later, the authors developed a similar system based on a different bacterial protein (RrgA), yielding the modules SnoopTag and SnoopCatcher (Figure 4D(i), right), which do not cross-react with SpyTag/SpyCatcher [55]. More recently, the group combined these systems with peptide sequences for cell adhesion (RGD) and MMP-sensitive motifs cleavage to produce a HA hydrogel [56]. SpyTag/SpyCatcher containing these peptide sequences were used for covalent crosslinking between HA chains, and SnoopTag/SnoopCatcher were used to introduce any ligand of interest, such as epithelial cell adhesion molecule (EpCAM) or E-cadherin (Figure 4D(ii)) [56]. Embedded fibroblasts were viable within these hydrogels and able to spread when RGD was present [56]. Non-malignant mammary cell spheres were also embedded in the hydrogels as the authors aimed at mimicking the interface between cancer cells and healthy cells by mediating the display of EpCAM [56]. EpCAM is a marker for cancer stem cells (CSC), and EpCAM positive breast cells are associated with CSC-like phenotype with increased metastatic ability [57]. Even though cells were viable, presentation of bound EpCAM led to the dissociation of the embedded mammary cell spheres, as opposed to hydrogels with no EpCAM [56]. Thus, this type of strategy can be a way to produce GAG covalent hydrogels and effectively present biomolecules of interest.

#### 2.1.4. Crosslinking with Synthetic Polymers

Different GAG derivatives have been used to produce hydrogels using synthetic polymers as crosslinkers (Figure 3D). Thiolated derivatives of HA [58,59], HEP [59,60], CS [58] have been synthesized for subsequent formation of hydrogels covalently crosslinked with PEG diacrylate (PEGDA). The use of PEG based crosslinkers in GAG based systems is an interesting option, since this polymer is not only biocompatible and bioinert but also of hydrophilic nature. Besides, PEG polymers exist commercially with a wide variety of molecular weights and functional groups, allowing versatile combinatorial strategies. Mechanical properties of produced hydrogels can be tailored not only by the degree of substitution of GAG derivatives but also by the molecular weight and concentration of crosslinkers. Kuang L et al. [58] studied how the effect of increasing GAG thiolation degree and crosslinker molecular weight impacted the formation and final properties of hydrogels composed of HA and CS (Figure 4E). Increasing HA and CS thiolation degree and PEGDA molecular weight significantly decreased gelation time from 30 min down to 5 min, concomitantly reducing swelling ratios and increasing storage moduli (G’) of HA/CS hydrogels [58]. The authors also evaluated how entrapped MSC reacted to changes in the hydrogel microenvironment, namely, matrix stiffening, by analyzing the activation of focal adhesion kinase (FAK) signaling pathways [58]. They observed that within a time span of approximately 30 min, with matrix stiffness ranging from 0.01 to 3.2 kPa, MSC were able to sense and respond to hydrogel stiffening [58]. The possibility of modulating intrinsic system parameters such as gelation rate, swelling, rheological and mechanical properties is important for fine-tuning hydrogels’ biological performance. For clinical application, for example, hydrogels should be easy to handle and apply/implant, and so the gelation gap should be within the range of time of clinical procedures. They should also provide enough mechanical and structural stability to endure such procedures without compromising their performance on host tissues.

#### 2.1.5. Reversible Crosslinking

##### Dynamic Covalent Crosslinking

**Figure 5:**
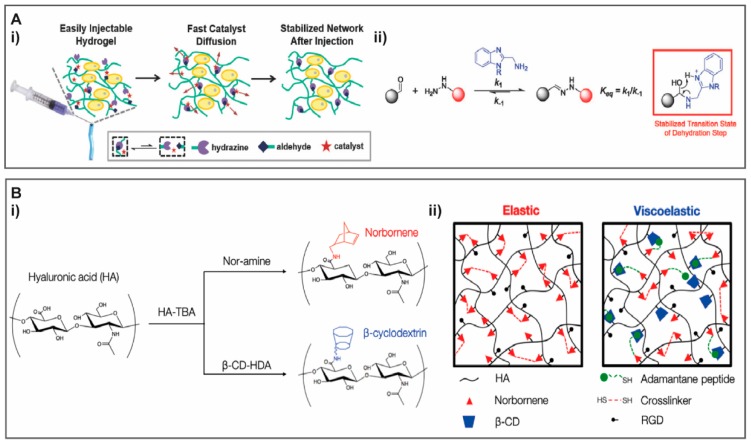
(**A**) (i) Modulation of dynamic properties of hydrazone crosslinks by catalyst in injectable hydrogel: upon injection, the catalyst promotes rapid exchange of hydrazone crosslinks and rearrangement of network that facilitate flow; after injection, the catalyst rapidly diffuses away to slow down hydrazone exchange, improving structural stability; (ii) catalyst-accelerated hydrazone equilibrium (catalyst in blue) [63]. (**B**) Example of GAG hydrogel based on cyclodextrin-based inclusion complexes with norbornene. (i) HA was derivatized to HA-TBA (tetrabutyl ammonium) salt and modified with norbornene groups (NorHA) or β-cyclodextrin groups (CD-HA). (ii) Elastic hydrogels produced with covalent crosslinks between norbornene groups were introduced using di-thiol crosslinkers via light-mediated thiol-ene addition; viscoelastic hydrogels, thiol-ene photochemistry was used to introduce supramolecular interactions between CD-HA and adamantane (guest) groups on thiolated peptides, besides the dithiol-mediated covalent cross-links between the norbornenes [72]. (Reproduced with permission from [63,72]).

Dynamic exchangeable covalent crosslinks are also being studied as an alternative to more traditional static chemical or physical crosslinking (Figure 3E). This can be achieved by hydrazone covalent crosslinks, formed between hydrazine and aldehyde groups, which are able to associate and disassociate in a structure-dependent rate [61]. Hydrazone crosslinked systems have been used for the production of 3D cell microenvironments [62], including with HA [40,63]. To improve hydrogel injectability and cell viability, Lou et al. [63] modified HA with hydrazine and aldehyde groups and incorporated a catalyst (Figure 5A(i)). The catalyst accelerated the formation and exchange of crosslinking bonds (Figure 5A(ii)) to facilitate hydrogel injection, but was readily diffused thereafter, leading to a lower exchange rate that promoted stabilization of the hydrogel structure [63]. The authors were able to modulate gelation time, storage and loss moduli as well as stress relaxation rate, by varying catalyst or polymer concentrations [63]. Besides, incorporation of catalyst in the formulation significantly increased endothelial cell viability post injection [63]. Incorporation of coumarin derivatives can also be explored for reversible covalent GAG systems, as they can be photo-cleaved when irradiated under UV light (approximately 250 nm) and near infrared light [64].

##### Inclusion Complexes

GAG derivatives can be produced by including hydrophobic groups to modulate their hydrophilic nature, providing a GAG amphiphilic character that can be suitable for particular applications, such as the delivery of hydrophobic compounds. Hydrogel formation of amphiphilic HA relies on supramolecular self-assembly, with establishment of physical bonds, namely, hydrophobic interactions between polymer chains [65]. Reported HA amphiphilic derivatives comprise the incorporation of riboflavin [66], hexylamine [65], dodecylamine [65] or octadecylamine [65,67,68].

The inclusion of hydrophobic domains can be explored to develop self-assembly hydrogels with high affinity bonds and controlled extent of self-assembly complexes. Self-assembly supramolecular GAG hydrogels with azobenzene and β-cyclodextrin HA derivatives have been reported (Figure 3F) [69]. Cyclodextrins are oligosaccharides that form reversible host-guest complexes with particular compounds, in a dimensional and geometric dependent manner, being widely used in the design of delivery systems with controlled and targeted release [70]. Cyclodextrins form cavities with hydrophobic interiors and hydrophilic surfaces interacting with their guest compound in self-assembly processes, occurring mainly via hydrophobic interactions but also via van der Walls and dipole-dipole interactions [71]. In the particular case of Rosales AM et al. [69], supramolecular crosslinking was induced or reverted by exposure to light.

Using cyclodextrin and norbornene HA derivatives, Hui E et al. [72] studied the effect of hydrogel viscoelasticity and stiffness by independently modulating these parameters. Viscoelastic hydrogels were produced by norbornene-HA reaction with adamantane thiolated peptides and dithiol crosslinker (dithiothreitol), which enabled supramolecular interactions with cyclodextrin-HA interactions via the adamantane (guest) group (Figure 5B(i–ii)) [72]. The combinatorial crosslinking approach allowed the production of viscoelastic hydrogels (Figure 5B(ii)), with a different mechanical profile as compared to covalent norbornene-HA hydrogels that are purely elastic [72]. That can be observed by differences in loss moduli (G’’) of hydrogels with similar G’, with covalent hydrogels presenting significantly lower G’’ than hydrogels with covalent and physical crosslinks [72]. Differences were also observed in stress relaxation, where norbornene-HA covalent hydrogels presented no stress relaxation, as opposed to hydrogels produced with norbornene-HA and cyclodextrin-HA, explained by the reorganization of physical cyclodextrin-adamantane host-guest bonds [72]. Such differences in the mechanical profile of hydrogels also impacted spreading and morphology of cells seeded in these hydrogels, showing a dependence of these parameters not only on stiffness but also viscoelasticity [72]. Such approaches envisage the increasing development of biomaterials with greater control of separate mechanical features, namely, the elastic and viscous character that differently affect cellular response.

### 2.2. Modulating Hydrogel Network Degradation

**Figure 6:**
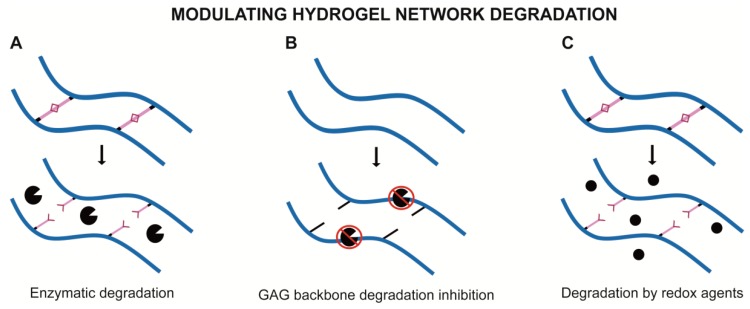
Schematic representation of main strategies to modulate network degradation of GAG-base hydrogels. To modulate network degradation, strategies can include enzyme sensitive sequences within crosslink bonds (e.g., MMP-sensitive peptide sequences) (**A**), or, oppositely, degradation can be inhibited at the level of GAG backbone by incorporation of functional groups that reduce GAG susceptibility to enzymatic cleavage (**B**). Crosslinking strategies can also be designed to be sensitive to redox agents (**C**).

Besides controlling hydrogel network formation, it may also be of interest to modulate GAG hydrogel degradation, for instance, to mimic the dynamic turnover processes occurring during ECM remodeling. This can be achieved by introducing stimuli-responsive crosslink bonds or new functional groups that modulate GAG sensitivity to cleavage (Figure 6). One example is the incorporation of peptide sequences sensitive to enzyme activity within the crosslinking bonds (Figure 6A). Following on the study described above in this section, in their HA-transglutaminase based scaffold, Broguiere et al. [39] introduced a matrix metalloproteinase (MMP)-sensitive sequence within one of the transglutaminase peptide sequences used (Figure 4B), enabling cell-driven hydrogel biodegradation by the action of MMP produced by cells [39].

On the other hand, hydrogel degradation can also be designed by incorporating new chemical groups that decrease GAG susceptibility to enzymatic degradation (Figure 6B). Pavan M et al. [73] studied enzymatic degradation susceptibility to human MMP and hyaluronidases of HA derivatives with different alkyl group lengths and degree of substitution. The authors were able to inhibit HA degradation by these enzymes when incorporating an hexadecyl moiety [73]. Materials such as this can be developed for administration in pathological scenarios such as osteoarthritis or chronic wounds that are, amongst others, defined by unbalanced inflammatory events frequently involving exacerbated amounts of MMP [74,75].

Covalent bonding can also be designed to be susceptible to physicochemical conditions, such as redox environment (Figure 6C). Gao Z et al. [76] reported the development of a HA-based system covalently crosslinked with aminoethyl disulfide crosslinkers, which are sensitive to reducing agents (e.g., glutathione). This type of stimuli-responsive systems can be relevant for applications under inflammatory conditions, where it is typical to observe oxidative stress caused by unbalanced production of reactive oxygen and nitrogen species [77].

Overall, GAG and their derivatives can successfully be used as the single component of hydrogels for in vitro and in vivo applications, either in the form of injectable materials or 3D scaffold. Still, for particular purposes, it may be of interest to combine GAG with materials of distinct nature and properties, as discussed in the following section.

## 3. Hybrid Hydrogels Containing GAG-Based Modules

Hydrogels containing GAG in combination with other polymers, herein referred as hybrid hydrogels, can be designed to more closely mimic the multi-component and multi-functional nature of the native ECM. The inclusion of GAG in hybrid systems is differently motivated depending on the GAG in question. Many systems include HA, not only because of its rheological and mechanical properties but also due to its biocompatibility and specific biological functions. On the other hand, the motivating factor for selecting CS or HEP for incorporating in hybrid systems is often related with their biological role, namely, the growth factor retention capacity. Additionally, hybrid systems can be used as platforms for controlled release or improved retention of GAG that, given their highly hydrophilic nature, are frequently difficult to retain within hydrogels if not efficiently bound or entrapped.

In this section, strategies involving the combination of GAG with other materials, namely, peptides, proteins and other polysaccharides, will be discussed.

### 3.1. Proteins and Peptides

#### 3.1.1. Collagen

Collagen hydrogels produced with GAG have been reported for cartilage regeneration [78,79,80], vocal fold tissue engineering [81], skin wound healing [82], pancreatic islet transplantation [83] and vascular applications [84], among others. Additionally, GAG have been combined with collagen hydrogels for growth factor presentation and release regulation, namely, for studying the effect of sulfation in such processes [85,86,87].

Collagen is one of the main components of mammalian ECM, particularly in tissues where high mechanical loadings are imposed, such as cartilage and bone. Collagen is composed of polypeptide chains of glycine, proline and hydroxyproline amino acids arranged in a triple helix conformation [88]. In vivo, different types of collagen can be distinguished, namely, fibril forming, which include collagen type I, II and III, and network-forming, such as collagen type IV [89]. Collagen gels are formed by the association of collagen polypeptide chains into fibers and fibrils that can form branched networks in a gelation process dependent on pH, temperature and ionic strength [90].

As referred above, hybrid hydrogels can be developed as carriers for delivering GAG. Nevertheless, collagen gels are characterized by poor mechanical performance and so interpenetrating networks (IPN) composed of collagen and GAG can be physically or chemically crosslinked to improve GAG retention. For meniscus tissue applications, Koh RH et al. [78] modified HA with hexamethylenediamene to produce crosslinked HA combined with collagen in a riboflavin-containing system. Riboflavin promotes collagen interhelical crosslinking under UV light exposure, increasing stiffness and decreasing swelling ratio of produced hydrogels [79]. Concomitantly, the inclusion of crosslinked-HA within riboflavin-crosslinked collagen hydrogels led to a significant increase of elastic moduli of these hybrid hydrogels, even at small quantities (1 % *w*/*v*) [79]. This demonstrates the dual role (biological and structural) of incorporated HA.

**Figure 7:**
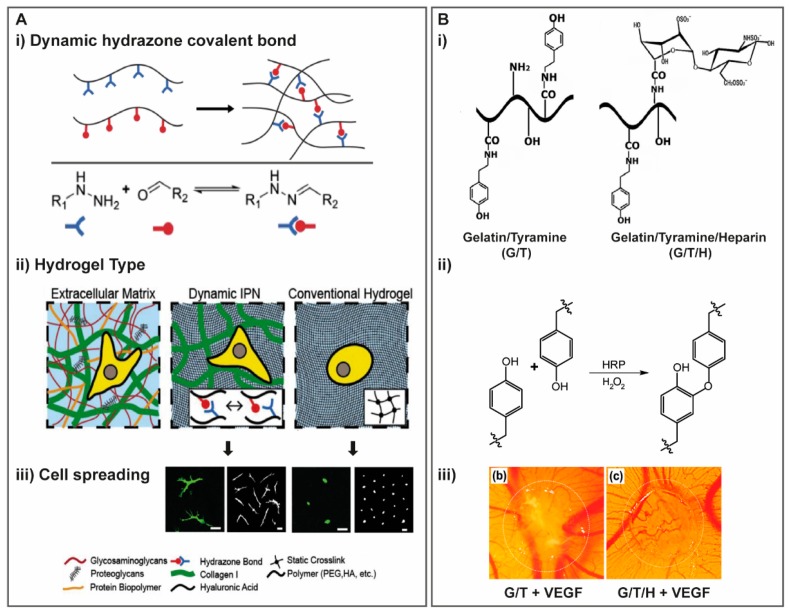
(**A**) HA/collagen IPN developed to mimic fibrillarity and viscoelasticity of the ECM. (i) Scheme of formation viscoelastic HA single network hydrogel via dynamic covalent hydrazone crosslinking and chemical structures of modified HA; (ii) representation of composition and network structure of ECM, dynamic HA-collagen IPN and conventional statically crosslinked hydrogel. (iii) MSC spreading is more pronounced in these dynamic IPN hydrogels with higher stress relaxation in comparison with more conventional hydrogels that have very low stress relaxation. [91]. (**B**) (i) Chemical structure of gelatin/tyramine (G/T) derivative and gelatin/tyramine/HEP (G/T/H) hydrogels; (ii) crosslinking mechanism by HRP and H_2_O_2_ addition to form injectable G/T/H hydrogels and (iii) comparison of new blood vessel formation of G/T and G/T/H hydrogels using an in vivo chicken chorioallantoic membrane (CAM) assay and after 5 days of incubation [103]. (Reproduced with permission from [91,103]).

Collagen-GAG combinations have been used as platforms to study the biological response to hydrogel stiffness and stress relaxation to better recapitulate native ECM mechanical properties. An IPN hydrogel composed of HA and collagen I has been used to study stress relaxation events and its impact on cell behavior [91]. The strategy was based on modifying HA to contain hydrazine or aldehyde groups, optimized to produce a dynamic crosslinking where hydrazone covalent bonding concomitantly formed and cleaved (Figure 7A(i,ii)) [91]. Interestingly, the authors were able to modulate the formation/cleavage rating of hydrazone bonds to obtain hydrogels with similar storage moduli but faster or slower crosslink exchange and so faster or slower stress relaxation, respectively [91]. Moreover, two different stages of stress relaxation were observed: Over a short time period, a fast relaxation seemed to be driven by collagen fiber realignment, followed by a slower relaxation attributed to the slower dissociation process of the hydrazine crosslinks in HA [91]. The authors then compared differences in cell behavior on static and dynamic hydrogels, including hydrogels with different stress relaxation rates [91]. There was an increase in cell spreading, formation of focal adhesions and fiber realignment in dynamic, stress-relaxing hydrogels, as opposed to static (no covalent rearrangement) hydrogels [91]. Cell spreading was even affected by differences in stress relaxation rates, significantly increasing in faster relaxing hydrogels (Figure 7A(iii)) [91].

GAG derivatives with higher ability to physically interact with collagen can also enable the production of hybrid hydrogels with improved mechanical properties. In an effort to recapitulate the dynamic macromolecular interactions occurring at the ECM, Federico S et al. [92] produced a HA derivative containing peptides derived from decorin, a CS/DS PG reported to have a role in collagen microfibril formation [93]. The authors modified HA backbone with collagen-binding peptides (LSELRLHNN) from decorin, thus promoting reversible, dynamic physical interactions between collagen and HA [92]. This HA modification led to an increase in G’ of more than an order of magnitude in collagen/modified-HA gels when comparing to collagen/non-modified HA or collagen alone, which is indicative of supramolecular network formation in the newly designed system [92]. Additionally, the authors observed that fibril diameter was reduced when modified-HA was used, which was expected due to the known role of decorin in collagen fibril diameter regulation [92]. The authors were thus able to modulate G’ of produced hydrogels, while better replicating some functional and structural ECM features [92].

Covalent GAG hybrid networks were already used as retention mechanism in collagen/GAG microcarriers. Hydrogels composed of collagen with either sulfated HA or CS were produced for growth factor retention/release, but entrapped GAG was poorly retained, with less than 7% GAG content remaining after 1 h of incubation [87]. To overcome this issue, Roth S et al. [86] produced hydrogels composed of acrylated-HA and collagen I, to embed microgels of collagen I and sulfated HA. Since sulfation and acrylation are incorporated via the HA carboxylic groups, they cannot be performed simultaneously with high yields. Thus, this strategy allowed the improvement of sulfated HA retention, while allowing its incorporation within a collagen artificial matrix [86].

#### 3.1.2. Gelatin

HA [94,95,96], HEP [97] and CS [98,99] have been combined with gelatin for bioprinting [100,101,102] and delivery systems [101,103] and for applications in wound healing [98,101,104], hemorrhage control [105], vascular [103,106] and cartilage tissue engineering [94,97]. Gelatin is obtained from collagen denaturation processes and, like collagen, it undergoes temperature and time dependent gelation [107,108], with resultant gels being characterized by poor mechanical properties. This has motivated the development of gelatin derivatives [109,110] for the production of covalent hydrogels with improved mechanical stability [111].

Incorporating GAG in gelatin scaffolds can also modulate cellular response. In gelatin/HA scaffolds, improved MSC differentiation into the chondrogenic lineage was observed in scaffolds with higher HA content, even though cell viability was similar in all formulations [112]. A gelatin-tyramine (G/H) derivative covalently bound to HEP (G/T/H, Figure 7B(i–ii)) was produced to improve vascular endothelial growth factor (VEGF) release and in vivo neovascularization [103]. VEGF loaded G/T/H hydrogels increased cell infiltration and angiogenesis in vivo [103]. Implantation of VEGF-loaded G/T/H hydrogels led to a significantly higher number of new blood vessels when comparing to the control and VEGF-loaded G/T hydrogels in in vivo CAM assays (Figure 7B(iii)) [103]. Similar results were obtained for VEGF-loaded G/T/H hydrogels implanted subcutaneously, which presented significantly higher number and area of new blood vessels than the other groups (G/T, G/T/H and VEGF-loaded G/T) [103]. Curiously, G/T/H hydrogels alone also presented some new vessels surrounding the gel and a few extending inside it [103].

The chosen method for GAG incorporation in protein hydrogels can impact both cell response and hydrogel physical properties. Brown et al. [97] studied the effect in HEP bioactivity (clot formation and growth factor interaction) of two different modifications, methacrylation and thiolation, for incorporation in methacrylated gelatin hydrogels. The authors observed that thiolation better preserved bioactivity when compared to non-modified HEP, but methacrylation led to higher HEP retention rates when compared to thiolated HEP, for the same initial GAG concentration [97]. As expected, cell proliferation, chondrogenic differentiation and ECM deposition were improved in hydrogels composed of thiolated HEP as compared to methacrylated HEP [97].

#### 3.1.3. Silk Fibroin

Silk fibroin is a fibrous protein extracted from silks produced by a wide variety of arthropods. For biomedical applications, it is mainly obtained from cocoons of Bombyx mori silkworm [113].

Silk fibroin hydrogels are formed from their transition from amorphous state to β-sheet conformations, which makes hydrogels strong but brittle. The combination of silk fibroin with GAG can be a strategy to improve and modulate the brittleness of produced hydrogels [114]. Silk fibroin has been combined with GAGs, mainly HA [115,116,117], with or without other polysaccharides such as alginate [118,119] or chitosan [120], for applications in skin wound healing [116,119], vascular growth factor delivery [121] and cartilage tissue [122]. HA has been reported to improve mechanical properties of silk fibroin scaffolds partially by supporting silk fibroin transitions to β-sheet conformations [114,123], in a pH dependent complexation mechanism, and by leading to the formation of crystalline and non-crystalline phases [124].

While there are silk fibroin/GAG systems relying solely in physical interactions [121], covalently crosslinked hydrogels have also been reported, including enzymatically crosslinked silk fibroin/HA hydrogels [125]. A multicomponent silk fibroin/gelatin-CS-HA covalently crosslinked by carbodiimide chemistry has also been reported for cartilage tissue engineering [99].

#### 3.1.4. Self-Assembly Peptides

**Figure 8:**
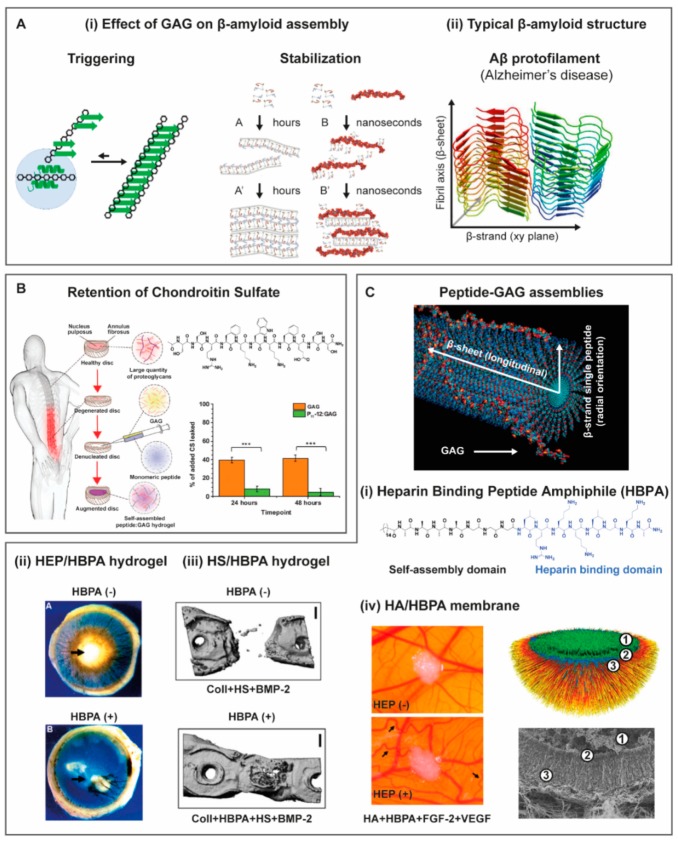
(**A**) (i) Schematic representation of GAGs interference on peptide self-assembly, either triggering formation or stabilizing preformed β-amyloid fibrils. (ii) Typical crystalline structure of Alzheimer’s disease Aβ plaques. Peptide β-strand segments orient parallel to the fibril cross-section (xy plane) and interact laterally to form long β-sheets aligned with the fibril long axis. Reproduced/adapted from [129,130] (i) and [128] (ii), with permission. (**B**) Positively charged, serine-rich peptide sequence bearing a pattern of alternating charged/polar and aromatic amino acids. CS induces self-assembling into β-sheet tapes improving GAG retention and restored compressive stiffness in an ex vivo model of denucleated intervertebral discs. Reproduced/adapted from [138], with permission. (**C**) Schematic representation of a peptide amphiphile (PA) self-assembling into nanofibers, which surface binds GAG molecules. Nanofiber cross-section shows PAs β-strands distributed radially and β-strands interacting laterally to form β-sheets aligned with the nanofiber long axis. (i) Chemical structure of a PA bearing a heparin binding sequence (HBPA). (ii) Hydrogels formed by HBPA and HEP showed FGF-2/VEGF retention, improving angiogenesis in a rat cornea model. (iii) Hydrogels formed by HBPA and HS showed BMP-2 retention and improved bone healing. (iv) Membranes formed by self-assembly of HBPA with HA and HEP showed FGF-2 and VEGF retention and enhanced angiogenesis in a CAM model. Membranes showed a hierarchical structure comprising a HBPA hydrogel region (1), a permeable membrane (2) and perpendicular nanofibers formed by GAG/peptide electrostatic interactions (3). (Reproduced with permission from [146] (i and ii), [153] (iii), [156,157] (vi)).

**Scheme 1:**
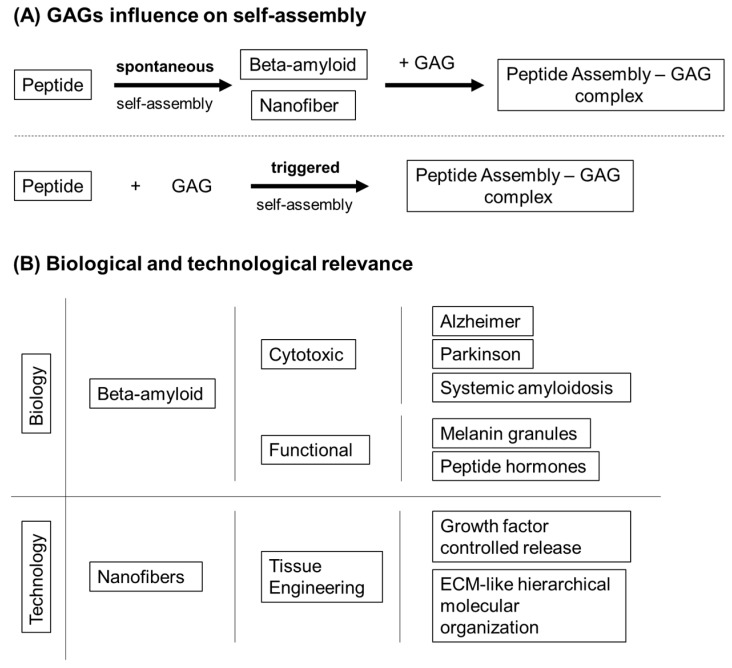
(**A**) GAGs can influence peptide self-assembly through two different general mechanisms, i.e., either nucleation or stabilization of self-assembled structures. (**B**) Interaction with self-assembling peptides has both biological and technological implications, since de novo designed peptide self-assembly systems retain structural hallmarks of beta-amyloids.

The combination of GAG with peptide self-assembly has high biological and technological significance (Scheme 1). GAG have been found to interfere with the mechanism and stability of peptides that self-assemble into either functional or cytotoxic beta-amyloid aggregates (Scheme 1A, Figure 8A(i)). Similarly, the de novo design of GAG-interacting peptide self-assembly systems is paving the way to create hydrogels for tissue engineering applications with improved growth factor controlled release and mimicking the ECM hierarchical molecular organization (Scheme 1B).

Degenerative diseases such as Alzheimer, Parkinson and systemic amyloidosis are associated with self-assembly of misfolded proteins and natively unfolded peptides into amyloid fibrils (Scheme 1B) [126,127]. The polypeptide chains assume a β-strand conformation that runs parallel to the fibril longitudinal axis, resulting in highly ordered, insoluble aggregates that accumulate in the afflicted tissues (Figure 8A(ii)) [126,127,128]. Interestingly, amyloid fibrils can also have a biological function, such as the storage of peptide hormones [129,130] and formation of melanin granules (Scheme 1B) [131]. In vivo, amyloid fibrils usually associate with GAG (Figure 8A(i)) [126,127]. In the case of intrinsically amyloidogenic peptides, this association affects the kinetics and mechanism of self-assembly [127,130]. Strikingly, the presence of GAG can trigger the formation of amyloid fibrils from non-aggregating peptides [132,133] or of low aggregation propensity (Scheme 1A) [129]. The biological and pathological relevance of the association of GAG with amyloid fibrils is still a matter of debate, but it has immediate implications for the design of self-assembling biomaterials.

Hydrogels based on self-assembling peptides are mostly comprised by fibrils with nanoscale lateral dimensions. The elongated shape of these fibrils is typically determined by peptide propensity to assume a β-strand conformation, leading to the formation of β-sheets, which hydrogen bonding propagates along the longitudinal axis of the fibril (Figure 8C) [134,135,136,137]. Such mechanism is highly reminiscent of amyloid fibril formation (Figure 8A(ii)), but remarkably, de novo designed peptide self-assembly systems are generally biocompatible [134,135,136]. For this reason, together with the facility to decorate the nano-assemblies surface with biochemical signals by simply extending the amino acid sequence with bioactive peptides, this type of hydrogels has been widely proposed as scaffolding biomaterials for TE [134,135,136].

GAG-interacting systems with self-assembling peptides have also been designed to take advantage of GAG role in mechanical properties, bioactivity and triggers for self-assembly and gelation. Miles et al. [138] developed self-assembling peptides that increased GAG retention and restored compressive stiffness in an ex vivo model of denucleated intervertebral discs (Figure 8B). By making rational mutations to peptides bearing a pattern of alternating charged/polar and aromatic amino acids known to self-assemble into β-sheet tapes, [139] they found that positively charged serine-rich peptides enhanced interactions with CS, triggering gelation, improving retention and restoring compressive stiffness [138]. They suggested that these hydrogels could be used for the regeneration of the PG-rich nucleus pulposus, where loss of GAG, predominantly based on CS, is associated with intervertebral disc degeneration.

Growth factor binding affinity to extracellular GAG restricts diffusion, contributes to formation of concentration gradients, assist on colocalization of growth factors and receptors, and protects against growth factors degradation [140,141,142]. For instance, bone morphogenetic protein 2 (BMP-2) [143], VEGF [144] and fibroblast growth factor (FGF) [145] have binding affinity to HEP and HS. For this reason, GAG have been used in conjunction with self-assembling peptides for the controlled release of growth factors in tissue engineering applications (Figure 8C). For instance, Rajangam et al. [146] designed a peptide amphiphile (PA) that self-assembles into cylindrical nanofibers, with a HEP binding peptide (LRKKLGKA) [147] at the surface (Figure 8C(i)). HEP binding PA (HBPA) solutions did not form hydrogels alone, but gelation could be triggered by HEP. Formed hydrogels exhibited prolonged release of VEFG and FGF-2. When using concentrations of these growth factors that by themselves did not induce any detectable angiogenesis, the hydrogel delivery system induced substantial vascularization in a rat corneal angiogenesis model (Figure 8C(ii)), [146] showing HBPA nanofibers combined with HEP harnessed GAG functional role in regulating and amplifying growth factor signaling. A control PA with a scrambled sequence presented a similar gelation behavior. Hydrogel formation was also triggered by HEP, presumably due to electrostatic charge screening [148]. Both scrambled and native HBPA sequences hold the same positive net charge. Remarkably, hydrogels comprising the scrambled sequence showed significantly less in vitro angiogenic bioactivity [148]. Fast vascularization of transplanted pancreatic islets is crucial for β-cell survival and, therefore, for islet engraftment with preservation of insulin secretion. In typical transplantation procedures, revascularization requires about two weeks, compromising viability and function. Delivery of VEGF and FGF-2 from HEP/HBPA hydrogels adjacent to transplanted islets significantly increased blood vessel density in the mouse omentum, enhancing islet engraftment and successful restoration of normoglycemia [149]. Isolated HBPA nanofibers decorated with HEP were also formed at concentrations 100 times lower than that required for gelation. Islets perfused with the HEP-binding nanofibers increased in vitro islet survival and insulin secretion and, when combined with VEGF and FGF-2, enhanced sprouting of intra-islet endothelial cells [150]. HEP/HBPA hydrogels were also used to capture and amplify the paracrine environment created by MSC during native tissue repair. Injection of hydrogels formed with conditioned medium obtained from murine MSC cultured under hypoxia conditions improved cardiac function in infarction-perfusion and hind limb ischemia mouse models. This was attributed to improved retention and activity of MSC secreted growth factors [151].

HS showed angiogenic properties similar to HEP when bound to HBPA. Hydrogels formed by the interaction between HS and HBPA were injected subcutaneously in a mouse model, yielding de novo vascularization and physiological microcirculation without increased permeability or persistent inflammation. These results were corroborated by dynamic observations in a murine dorsal skinfold chamber, supporting the potential of using these materials in chronic wound healing and tissue engineering applications [152]. In bone, BMP-2 binds to HS at transmembrane glycoproteins such as syndecan, facilitating colocalization with BMP-2 receptors [141]. For this reason, HS/HBPA hydrogels, formed within the pores of a collagen sponge, were also used to deliver BMP-2 at concentrations one order of magnitude lower than that required for healing in a critical-size femoral defect rat model. The delivery approach resulted in an improved bridging of bone defects and enhanced the formation of mature bone (Figure 8C(iii)) [153], showing how self-assembling peptides used in conjunction with GAG can amplify the regenerative capacity of growth factors.

Capito et al. [154] found that a membrane is rapidly formed at the interface of aqueous solutions of cationic peptide amphiphiles (PA) and HA. These membranes were robust enough to allow suturing, permeable to proteins and self-healing. Remarkably, the membranes presented a hierarchically ordered structure, comprising long nanofibrils, containing both HA and PA, aligned perpendicular to the membrane surface and spanning the entire membrane thickness [154]. Computer simulations and experimental validation showed that the formation of hierarchical membranes requires strong interactions between molecular components at early time points in order to rapidly generate a diffusion barrier between both solutions [155]. Orthogonal alignment of nanofibrils was only observed for β-sheet forming PAs that assemble into nanofibers with a high surface charge density [155]. Membrane-confined compartments can be formed injecting a HA solution into a PA solution, the content of which can later be transformed into a hydrogel. PA hydrogels formed in this way sustained MSC viability up to four weeks and further supported their chondrogenic differentiation, showing that the high permeability of these hierarchical membranes provides sufficient diffusion of nutrients and biochemical signals [154]. Membranes could also be formed at the interface between solutions of HEP binding HBPA and HA, owing to HBPA inherent cationic character but no hierarchical organization was observed. Interestingly, the membrane hierarchical organization was recovered adding HEP to the HA solution (Figure 8C(iv)) [156,157]. Planar membranes containing HEP sustained the release of FGF-2 and VEGF for up to two weeks in vitro, which showed enhanced angiogenesis in a CAM assay (Figure 8C(iv)) [156]. The authors suggest that this type of membranes, which can be formed in situ to cover arbitrary areas of tissue and deliver therapeutic proteins, could be used as dressings for chronic wounds or for cell transplantation in regenerative medicine applications.

### 3.2. Non-Mammalian Non-GAG Polysaccharides

#### 3.2.1. Chitosan

Chitosan is one of the non-mammalian natural polysaccharides more frequently combined with GAG. Obtained from the deacetylation of chitin found in crustaceous, insects and fungal walls, chitosan is composed of d-glucosamine and *N*-acetyl-d-glucosamine units, thus being of polycationic nature. Given its biocompatibility, chitosan is widely used in pharmacological formulations and applications in tissue engineering, particularly for bone tissue. CS [158,159,160,161], HA [160,162,163], HEP [164], HS [161] and DS [158,159,160] have been combined with chitosan and proposed for skin wound healing [160], cartilage [158,159], bone [162] and vascular [164] applications.

Chitosan-GAG membranes were used to test the hypothesis of a synergistic effect of co-immobilizing different GAG (CS-A, CS-C, DS and HEP) by Chen et al. [165]. The authors observed differences in chondrocyte morphology and proliferation, collagen and GAG production and gene expression of cells cultured with different GAG combinatorial concentrations [165].

Similarly, the effect of GAG presentation or delivery strategy was also assessed regarding biological outcomes by Uygun et al. [166] in chitosan membranes with ionically or covalently immobilized HEP, HS, CS, DS or HA, separately. The authors observed significant differences on MSC morphology depending on the GAG immobilization approach [166]. Surfaces with ionically immobilized GAG did not promote cell spreading even after 7 days, as opposed to surfaces with covalently-immobilized GAG [166]. Cell spreading was also dependent on GAG type and density, with immobilized HS and HA leading to higher cell spreading than other GAG, for the same density [166]. Interestingly, the authors also evaluated the ability of each surface to adsorb serum proteins (fibronectin and vitronectin), and for all GAG, the amount of protein adsorption increased proportionally to the amount of immobilized GAG [166]. This is an important aspect since cell interactions with biomaterials are known to be dependent on protein adsorption events.

Some chitosan-based systems rely on the possibility of forming polyelectrolyte complexes (PEC) with other charged materials, including sulfated GAG. For example, CS and chitosan physical complexes are formed between the positive charges of chitosan amine groups and negative charges of CS sulfate group [167,168,169]. Such chitosan/CS scaffolds can be produced at particular pH ranges and present pH-dependent properties, such as swelling and CS release [170]. Chitosan/CS PEC have been developed as a method for cell encapsulation and delivery with the ability to induce MSCs chondrogenic differentiation [168] and as vehicle for growth factor delivery [171]. For neovascularization applications, chitosan dibasic derivatives were also produced to promote electrostatic interaction with HEP and increase GAG loading into the scaffold [164].

**Figure 9:**
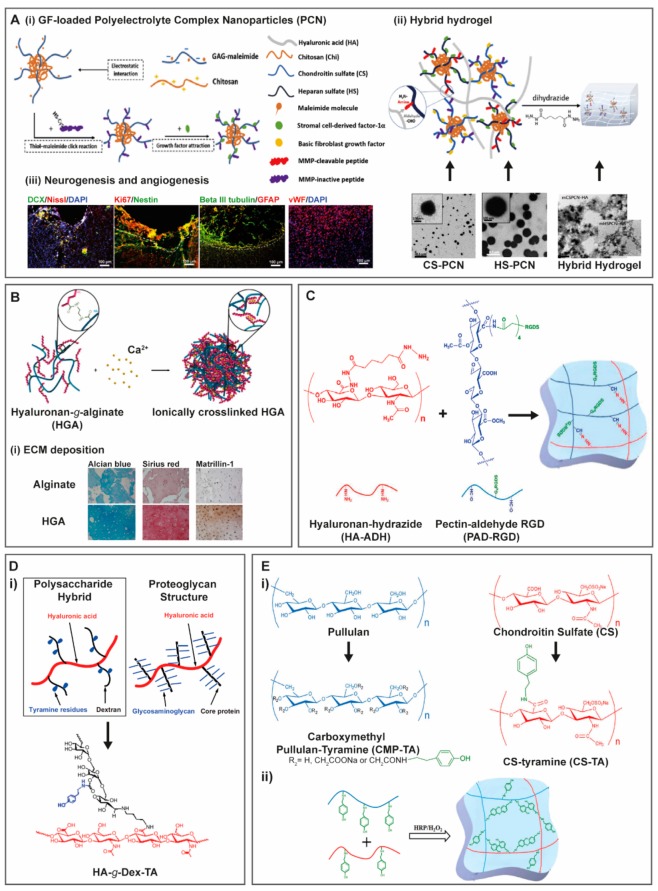
(**A**) (i) Formation of growth factor-loaded polyelectrolyte complex nanoparticles (PCN) through electrostatic interactions with chitosan and PCN coupling with MMP-sensitive/inactive peptide sequences via thiol-maleimide coupling reaction. bFGF and SDF-1a were sequestered on CS-PCN and HS-PCN, respectively; (ii) PCN covalent incorporation within HA-aldehyde hydrogel network through the amine terminated peptides on PCN and the aldehyde groups on HA and hydrogel was covalent crosslinking via hydrazone bonding; TEM images of CS-PCN, HS-PCN and hybrid hydrogels with CS-PCN and HSP-PCN (inset) within HA matrix; (iii) immunohistochemistry results of coronal sections of infarcted brain stained with doublecortin (DCX, migrating neural precursor cells), Niss1 (neuronal cells), DAPI (nuclei), Ki67 (cell proliferation) and Nestin (neural stem/progenitor cells) beta III tubulin (immature neurons), glial fibrillary acidic protein (GFAP, astrogliosis) indicating neurogenesis and with von Willebrand factor (vWF, endothelial cells) indicating angiogenesis (21 days after treatment) [161]. (**B**) Schematic representation of hyaluronate-*g*-alginate (HGA) hydrogel formation and (i) histological images of tissue after six weeks of hydrogel transplantation with primary chondrocytes showing increased ECM deposition in HGA samples than in alginate, indicated by higher intensity of Alcian blue, Sirius red and Matrillin-1 protein staining [184]. (**C**) Chemical structure of HA-hydrazide (red) and RGD-functionalized pectin-aldehyde (blue) and hydrogel crosslinking strategy [200]. (**D**) (i) representation of the chemical structure of the polysaccharide hybrid based on hyaluronic acid and dextran-tyramine (HA-*g*-Dex-TA) and comparison with structure of proteoglycan [202]. (**E**) Chemical structure of pullulan and CS and their corresponding derivatives, carboxymethyl pullulan-tyramine (CMP-TA) and CS-tyramine (CS-TA) and covalent hydrogel formation using these derivatives [217]. (Reproduced with permission from [161,184,200,202,217]).

Combinatorial approaches of PEC/covalent hydrogels have also been reported using different GAG derivatives and chitosan. Chitosan PEC nanoparticles (PCN) were produced with HS and CS maleimide derivatives and loaded with stromal cell-derived factor (SDF)-1α or FGF-2, respectively (Figure 9A) as a growth factor vehicle for ischemic stroke scenarios [161]. To have a controlled release of growth factors, the maleimide groups in HS and CS were covalently bound to the thiol termination of MMP-cleavable and MMP-inactive peptides, respectively, by click chemistry [161]. These functionalized PCN were then covalently bond to aldehyde derivatized HA through the covalent conjugation of the amine terminal in MMP-peptide sequences, while the HA derivative itself also formed a covalent network formed via hydrazone bond (Figure 9A) [161]. The release of covalently bound PCN was observed to be dependent to enzymatic concentration, whereas non-bound PCN were rapidly released from the hydrogel [161]. Minding their potential application, these hybrid hydrogels were injected in infarcted brain tissue of a photothrombotic ischemia rat model [161]. Overall, results showed that using this hybrid hydrogel for the controlled release of SDF-1α or FGF-2 enhanced motor recovery and promoted neurogenesis and angiogenesis in infarcted tissue (Figure 9A) [161].

Direct covalent crosslink between GAG and chitosan is also possible by producing derivatives of both components, as it is, for example, the covalent crosslinking between aldehyde-containing CS and carboxymethyl chitosan by Schiff’ base reaction [172,173].

#### 3.2.2. Alginate

Alginate scaffolds containing GAG have been developed for nerve tissue regeneration [174,175], wound healing [176,177], cartilage [178,179,180], muscle tissue [181,182] and bone regeneration [183]. Alginate, a linear polysaccharide derived from marine algae, is composed of guluronic acid and mannuronic acid monomeric units. Alginate can undergo ionic gelation when in the presence of divalent cations (e.g., Ca^2+^), allowing the formation of non-covalent hydrogels under mild, biocompatible conditions. Being biologically inert, alginate can be incorporated in 3D hydrogels to improve mechanical properties without triggering cellular responses, being suitable as a “blank slate” material.

In fact, ionic alginate-based systems become convenient for developing injectable and in situ gelling biomaterials, including when in combination with GAG and without the need for prior chemical modifications. For larynx rejuvenation, Choi YH et al. [181] developed an injectable and in situ gelling alginate/HA hydrogel loaded with FGF-2. The straightforward strategy was based on mixing both polymers with the growth factor and CaSO_4_ for ionic crosslinking [181]. This IPN hydrogel was then injected in rat laryngeal muscle tissues and decreased fibrosis while promoting an increased cross-sectional area of muscle fiber and myogenic response [181]. Alginate has also been combined with CS in Sr^2+^ ionically crosslinked biomaterials where the molecular weight of the alginate was varied to study the impact of matrix structural differences on encapsulated chondrocytes [180]. By varying the alginate/HA ratio (4:1, 2:1 and 1:1 *w*/*w*), Ansari S et al. [175] observed a decrease in the elastic modulus concomitant with an increase in pore size for hydrogels with increasing HA content. This enabled the authors to study the effect of elasticity in different populations of dental-derived MSC and the impact of alginate/HA ratio in the release of β-nerve growth factor (β-NGF), previously loaded [175]. In fact, the presence of HA significantly promoted proliferation and neurogenic differentiation of encapsulated MSC in alginate hydrogels [175]. Thus, these alginate/GAG biomaterials provide simple, straightforward methods for optimizing the final structural and biofunctional properties of hydrogels such as the variation polymer molecular weight, polymer concentrations and ratio and type and concentration of divalent cations.

Alginate/GAG systems can also combine covalent and physical crosslinking methods. Alginate has been grafted to HA backbone as a strategy to develop HA-based ionically crosslinked hydrogels (Figure 9B) [184]. HA was previously modified with ethylenediamine to incorporate amine groups able to react with alginate carboxyl groups by carbodiimide reaction [184]. The produced copolymer was able to undergo ionic gelation in the presence of Ca^2+^ cations, and storage modulus and gelation time were dependent on alginate/HA ratio and alginate molecular weight [184]. Additionally, the suitability of this system for cartilage tissue regeneration was assessed in vivo. Hydrogels were injected with primary chondrocytes in the dorsal region of mice, and HA-*g*-alginate hydrogels showed improved ECM deposition (proteoglycans and collagen, Figure 8B(-i)), preservation of chondrogenic phenotype and gene expression (SOX-9, aggrecan and type II collagen), when compared to alginate.

A similar approach was reported for alginate grafted to HEP, in this case to develop a responsive system for the controlled release of TGF-β1 [185]. Hydrogels were ionically crosslinked with calcium sulfate and in the presence of iron oxide nanoparticles sensitive to applied magnetic fields [185]. On-demand growth factor release could be obtained in these ferrogels upon magnetic field application as iron oxide nanoparticles movement causes alteration or disruption of hydrogel microstructure. On the other hand, Zhang Y et al. [186] developed an in situ gelling alginate/HA hydrogel based on two types of covalent bonding: using oxidized alginate and modified HA containing thiol and hydrazide groups, covalent crosslinking formed via disulfide or hydrazone bonds between alginate and HA. These covalent alginate/GAG systems are a versatile strategy for dual- or multi-crosslinking hydrogels as they can be developed to enable the formation of covalent bonds of distinctive nature while still partially preserving alginate ability to ionic crosslink.

#### 3.2.3. Pectin

Pectin is a branched polysaccharide present in the cell wall of land plants and can be extracted from peel and pulp of fruits (e.g., orange, lemon, apple) [187]. Pectin has been explored for drug delivery [188,189], wound healing [190,191,192], cell encapsulation [193] and cancer therapy [188,194,195]. Pectin contains at least three covalently linked polysaccharide domains, the linear homogalacturonan (HGA) domain and the branched (or ‘hairy’) domains rhamnogalacturonan-I (RG-I) and rhamnogalacturonan-II (RG-II) [196]. Similarly to alginate, pectin can form gels in the presence of different divalent cations through ionic interactions [197,198,199].

Oxidized pectin has been conjugated with a HA-dihydrazide derivative by covalent crosslinking (Figure 9C) [200]. This combination allowed the modulation of compressive modulus, gelation time, swelling ratio and hydrogel degradation by adjusting HA/pectin weight ratio [200]. Increasing HA content led to a decrease in gelation time, an increase in G’, critical stress and compressive modulus and decreased the swelling ratio, most likely due to higher crosslinking densities and molecular weights of HA chains, as proposed by the authors [200]. Additionally, these hydrogels, which also contained cell adhesive peptides covalently bound to pectin, enhanced chondrocyte gene and protein expression, also affected by the pectin/HA ratio [200]. In this system, cell-matrix interactions distinctively occur with HA via CD44 receptors and with pectin-RGD via integrin receptors. Thus, pectin/HA ratio interferes not only on the mechanical and structural properties of the network but also on the presentation of such cell-interactive domains and their potential interaction with cells [200]. Equal pectin/HA content led to improved chondrocyte proliferation, chondrogenic gene expression and ECM production, most likely due to an appropriate crosslinking density formed within the network and to the contribution of RGD peptides for cell-matrix interaction [200].

#### 3.2.4. Dextran

The dextran family comprises branched homopolysaccharides composed of D-glucopyranose units and produced by lactic-acid bacteria. Dextran-GAG combinations are reported for application in vocal fold [201] and cartilage [202] repair and regeneration and drug delivery [203].

A supramolecular hydrogel using HA and dextran derivatives with molecular recognition was developed as a strategy to improve HA retention and mechanical properties. Chen et al. [204] modified HA with β-cyclodextrin and dextran with 2-naphtylacetic acid to produce host-guest hydrogels. By varying the number of inclusion complexes, through the amount of modified dextran in the formulations, the authors observed a decrease in pore size and swelling ratio, as well as an increase in G’ with increasing dextran content [204]. Additionally, the developed system was able to sustain high viability rates of entrapped cells [204].

Jin R et al. [202] developed hydrogels mimicking the native ECM, regarding HA association with PG, by grafting dextran into HA backbone (Figure 9D). Dextran was also modified with tyramine groups to further allow enzymatic-induced covalent crosslinking with proteins [202]. Increasing dextran concentration or number of tyramine groups significantly decreased gelation time and swelling ratio, while increasing storage modulus of the hydrogels [202].

A semi-IPN composed of a photocrosslinkable dextran-hydroxyethyl methacrylate (HEMA) derivative mixed with HA was produced for cartilage regeneration, allowing the optimization of rheological and mechanical properties for 3D bioprinting applications [205]. The rheological behavior was dominated by HA presence, and the elastic behavior of the blends was increased with increasing HA concentrations (2% to 6% HA for 10% dextran), allowing extrusion and filament shape preservation during printing [205]. On the other hand, elasticity of crosslinked hydrogels was not affected by HA concentration and was only dependent on the dextran-HEMA [205].

The ability of HEP to interact with positively charged molecules has been explored in a compound release system combining this GAG with a cationic, amine-containing derivative of dextran [203]. In this case, HEP interacted not only with positively charged molecules intended for release but also the vehicle molecules themselves (the cationic dextran derivative) [203]. The release profile of microspheres was then evaluated in the presence of protamine, a positively charged protein that inhibits HEP by forming electrostatic complexes with this GAG [203]. It was observed that compound release was possible to tune not only by the degree of dextran amination but also the dextran/HEP ratio, with increasing amounts of entrapped HEP leading to higher compound encapsulation efficiency [203]. The presence of protamine induced compound release by competition with dextran/HEP and compound/HEP complexes [203].

#### 3.2.5. Cellulose

Cellulose is a linear polysaccharide composed of repeating units of glucopyranosyl, being the most abundant polysaccharide in the world. Its wide availability, biocompatibility and biodegradability motivated its use and research in the biomedical field. Nevertheless, and despite its highly polar nature, cellulose is insoluble in water and in most organic solvents, where it forms intricate networks of inter and intramolecular hydrogen bonding, entropic effects and hydrophobic interactions [206,207]. Solubility can be improved, for instance, using ionic liquids mixed with aqueous or organic cosolvents [208] or by chemically modifying cellulose to produce derivatives with improved solubility.

Cellulose and cellulose derivatives have been used to improve mechanical properties of HA-based materials [209,210,211], namely, as wound dressings [211,212] and bioinks for bioprinting [213]. Domingues RMA et al. [209] developed a nanocomposite hydrogel composed of aldehyde modified cellulose nanocrystals and HA derivatives, containing either dihydrazide or aldehyde groups, as an injectable material for tissue engineering. In this particular case, the cellulose nanocrystals were incorporated in HA formulations for mechanical and structural reinforcement, and the effect of varying cellulose content was analyzed [209]. The incorporation of cellulose nanocrystals in HA hydrogels led to a decrease in pore size and an increase in stiffness, while promoting adipocyte spreading and proliferation, when comparing to HA hydrogels [209]. Luo P et al. [212] prepared composite hydrogels composed of oxidized hydroxyethyl cellulose covalently crosslinked with adipic acid dihydrazide modified HA. Thiolated derivatives of carboxymethyl cellulose and HA were used to produce injectable in situ crosslinkable hydrogels, by the formation of disulfide bonds, as a strategy for drug delivery systems [214].

#### 3.2.6. Pullulan

Pullulan is a biocompatible neutral linear polysaccharide composed of maltriose trimers and is mainly produced by Aureobasidium pullulans fungus. Pullulan and its derivatives have been applied in a wide variety of biomedical applications, including drug delivery, tissue engineering and medical imaging, as reviewed elsewhere [215].

Grafting of pullulan to HA (HA-*g*-Pu) at varying molar ratios has been reported as a strategy to produce films for wound healing with improved resistance to enzymatic degradation [216]. Slower rates of degradation were obtained for HA-*g*-Pu exposed to hyaluronidase as opposed to HA alone [216]. On the other hand, HA-*g*-Pu films presented higher porosity and supported higher cell density throughout culture time than films solely composed of Pu [216].

For cartilage tissue engineering, carboxymethyl pullulan-tyramine was combined with CS-tyramine to develop an injectable hydrogel able to undergo in situ enzymatic crosslinking (Figure 9E) [217]. In this case, CS was incorporated to improve the biological activity of the pullulan-based system towards chondrogenic cells [217]. The crosslinking strategy enabled the modulation of gelation time, mechanical properties and degradation time of hydrogels with varying polymer concentrations, pullulan/CS weight ratios, HRP and H_2_O_2_ concentrations [217]. In general, increasing CS led to a decrease in G’, compressive modulus and degradation time and to an increase in swelling ratio, most likely due to lower CS crosslinking densities, high hydrophilicity and more electrostatic repulson in comparison with formulations with higher pullulan content [217]. Concomitantly, CS incorporation led to increased chondrocyte viability and proliferation and collagen type II and aggrecan gene and protein expression by encapsulated chondrocytes were also upregulated in CS-containing formulations [217]. This may be due to the inherent CS biological activity and the increased swelling ratios in CS-containing formulations, which improve nutrient exchange and growth factor sequestering [217]. More recently, an in situ crosslinking of a pullulan and CS hydrogel has been also achieved by oxidizing pullulan and modifying CS with a adipic dihydrazide moiety [218].

### 3.3. Decellularized Extracellular Matrix (dECM)

Native ECM can undergo chemical and/or physical processing to remove all cellular components, while preserving non-cellular components as much as possible, giving rise to decellularized matrices (dECM). Given the tissue-specificity of ECM structure and composition, dECM provide biomaterial platforms that more closely resemble the acellular composition and structure of the native tissue, as compared to other scaffolding materials [219]. Nevertheless, decellularization processes can negatively impact both structural and biochemical features of the final dECM [220,221], and support materials might be needed to restore structural stability.

One example is the use of modified CS and HA containing sulfate-*N*-hydroxysuccinimidyl succinate (sulfo-NHS) groups to induce covalent bonding between sulfo-NHS and primary amines present dECM particles [222]. Incorporating sulfo-NHS-GAG significantly increased the G’ and gelation kinetics and allowed the production of hydrogels with mechanically distinct regions to replicate what occurs in particular native tissues (e.g., spinal cord) [222].

## 4. GAG-Inspired Biomaterials

**Figure 10:**
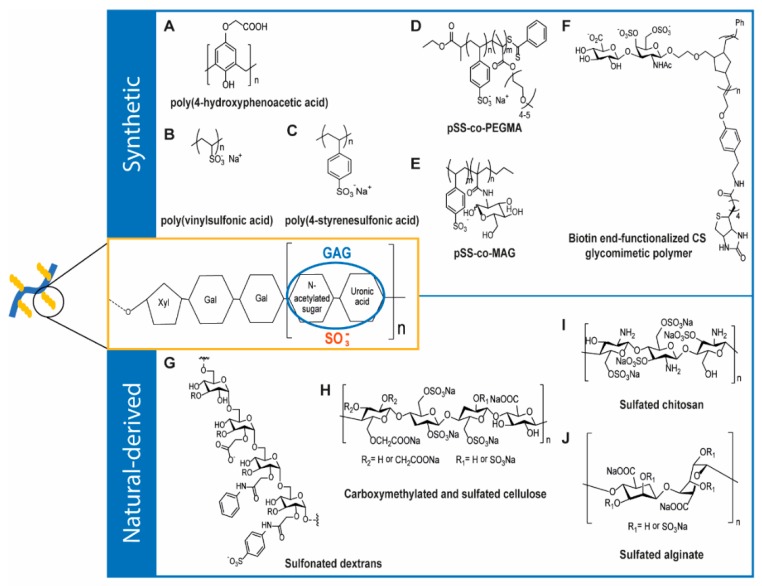
Synthetic (**A**–**F**) and natural-derived (**G**–**J**) GAG mimetic polymers. Approaches based on fully synthetic polymers comprise non-sulfated (**A**) or sulfated side chains (**B**,**C**,**D**). Alternatively, glycosidic units are displayed as side chains on synthetic polymer backbones (**E**,**F**). Approaches based on natural-derived polymers rely on the modification of polysaccharides backbone with sulfate groups (**G**–**J**).

As they are natural-occurring macromolecules, the application of GAG can be strongly limited by their structure heterogeneity, impurity and uncontrolled degrees of sulfation [223]. These drawbacks motivated research on chemically designed GAG-mimicking polymers, which present remarkable advantages, providing better control over sulfation patterns and purity. As a result, several compounds including synthetic and natural-derived polymers have been developed as potential GAG-mimicking systems (Figure 10). Amongst GAG, HEP has always been regarded as the “gold standard” GAG, due to its interesting anticoagulant and anti-inflammatory properties and ability to interfere with cell migration and proliferation [224]. As a result, most of the reported synthetic approaches target the synthesis of HEP-mimicking polymers.

### 4.1. Synthetic GAG-Like Polymers

Synthetic polymers have attracted special attention for developing GAG-mimicking materials as they allow fine-tuning of composition and structural properties. One of the first strategies to produce GAG-like synthetic materials involved the synthesis of linear polyaromatic non-sulfated anionic polymers, in which the negative charge arises from the presence of carboxylic groups in the polymerized phenol moieties. Among the library of compounds obtained by Regan et al. [225], through acid catalyzed polymerization of phenols with formaldehyde, the compound poly(4-hydroxyphenoacetic acid) showed HEP-mimicking ability (Figure 10A). It was able to inhibit proliferation of vascular smooth muscle cells (SMCs) induced by FGF-2, efficiently releasing FGF-2 and inhibiting heparanase activity [225,226]. In an attempt to better reproduce HEP structural features, polysulfonated compounds such as poly(4-styrenesulfonic acid) and poly(vinylsulfonic acid) have been synthesized by Liekens and co-workers [227] through free radical polymerization (Figure 10B–C). The compounds were able to bind FGF-2 by mimicking HEP interaction, and inhibit FGF-2-induced endothelial cell proliferation in angiogenesis and tumor growth [227]. Polysulfonate polymers have also been developed through reversible addition-fragmentation chain transfer (RAFT) polymerization of styrene sulfonate units with poly(ethyleneglycol)methacrylate (Figure 10D) [228]. The resultant polymer, poly(sodium 4-styrenesulfonate-co-poly(ethyleneglycol)methacrylate—pSS-co-pPEGMA) was able to bind the HEP binding domains of FGF-2 and VEGF [228,229].

Although the described strategies combined the linearity of the GAG backbone with the presence of pendant anionic groups, they did not include glycosidic moieties in the polymer structure. Thus, these compounds still exhibited large structural differences when compared to GAG. The fundamental role of the glycolic units was explored in an elegant work from Chen and co-workers [223], who synthesized a copolymer composed by 4-vinylbenzene sulfonate (SS) and 2-methacrylamidoglucopyranose (MAG) through RAFT polymerization (Figure 10E). They observed that polymers containing approximately 1:1 ratio sulfonate to glycol units promoted neural differentiation of embryonic stem cells (ESCs) in a performance similar, or even better than HEP [223]. Since it has been described that small oligosaccharide sequences may be responsible for the unique biological activities of the parent polysaccharides, investigation has been directed to the synthesis of GAG-like glycopolymers with enhanced synthetic control and reproducibility. In this kind of oligosaccharides, the carbohydrate density, as well as other physicochemical properties could be controlled through the choice of polymerizable saccharide and associated comonomers [230]. HEP-like compounds with controlled sulfation patterns and low polydispersity indexes have been obtained by Sun et al. [231] through the synthesis of fully sulfated lactose based glycopolymers. These polymers were synthesized by free-radical polymerization of acrylamide derivatized glycomonomers and exhibited anticoagulant activity [231]. In another interesting approach, Lee et al. [232] synthesized a new class of CS glycomimetic polymers that display defined sulfation motifs and are able to mimic the multivalent architecture of native GAG chains. The compounds exhibited a linear chain with pendant anionic disaccharide units and could be efficiently attached to surfaces, with specific orientation, consisting valuable tools for probing GAG functions, namely, for studying their interactions with proteins (Figure 10F) [232].

### 4.2. Natural-Derived GAG-Like Polymers

Some natural-derived polymers have also been explored as GAG-mimicking systems. While some naturally occurring polymers have been readily tested for their GAG-mimicking ability, others have been modified in order to better mimic structural features of GAG. Li et al. [233] reported the extraction and purification of a GAG-like polymer obtained from the marine gastropod abalone Haliotis discus hannai Ino. The polymer was composed by galactosamine, glucuronic acid, fucose and had a sulfation degree of 15.5%, expressing a GAG-like behavior through its high anticoagulant activity [233]. Fucose containing sulfated polysaccharides, known as fucans and/or fucoidans, have been widely investigated for biomedical applications, namely, due to their immunomodulatory, hemostatic, anti-inflammatory and antitumoral activity. Their ability to mimic the carbohydrate moieties of GAG encourages their application as scaffolds for delivery systems and tissue regeneration [234]. The potential of these polymers has also been demonstrated by Senni et al. [235], who extracted a fucoidan from brown algae, which showed ability to inhibit gelatinase A secretion and interleukin-1β on dermal fibroblasts in culture. Furthermore, it was found that this polymer increased the association rate of MMP with their specific inhibitors, being a potent modulator of connective tissue proteolysis [235]. The diversity of GAG-mimicking polymers that can be obtained from marine sources has been extended by Merceron et al. [236], who extracted a high-molecular weight sulfated polysaccharide produced by marine prokaryotes. Depolymerization and subsequent sulfation of this compound resulted in the synthesis of a natural-derived polymer with higher sulfation degree and ability to bind TGF-β1 more efficiently, which may be promising for driving efficient MSC chondrogenesis in cartilage tissue engineering [236].

Although these compounds have been readily extracted from marine sources, the first works on natural-derived polysaccharides as GAG-mimicking polymers have been carried out using commercially available dextran. Compounds with antithrombic activity have been developed through functionalization of crosslinked dextrans with carboxymethyl and benzylamide sulfonate moieties, introduced in the polymer backbone through carboxymethylation of hydroxyl groups and benzylamidation, followed by sulfonation, respectively (Figure 10G) [237]. Although GAG-mimicking dextran derivatives exhibited lower antithrombic activity than HEP, they revealed other important biological features such as anti-inflammatory, antibacterial, antiviral, regenerative and antitumoral activity. These GAG analogues revealed HEP-like ability in tissues such as skin, bone, colon, cornea and muscle [238]. More recently, polysaccharides with structure and bioactivity similar to HEP, based on regioselectively sulfated, carboxylated or carboxymethylated cellulose and sulfated chitosan have been synthesized by Groth and co-workers (Figure 10H) [239]. Biological activity of these compounds studied in C2C12 cells in the presence of bone morphogenesis protein-2 (BMP-2) revealed that some of them possess osteogenic activity, being potential candidates for application in bone tissue engineering [239]. The development chitosan-based GAG-mimicking polymers was also investigated by Ding et al. [240], who demonstrated that selective 6-O-sulfation of chitosan carried out in the presence of variable ratios of sulfuric acid and chlorosulfonic acid induced neural differentiation of embryonic stem cells (ESC) (Figure 10I). It was observed that the site of sulfation of GAG-mimetics plays an important role in the ability of the molecules to regulate stem cell differentiation and that a higher degree of sulfation increased levels of βIII-tubulin expression in ESC [240]. The linear conformation of alginate polysaccharide allied to its ease modification encouraged the synthesis of alginate-based GAG-mimicking polymers (Figure 10J). Such polymers could be obtained by esterification of hydroxyl moieties using different sulfation agents, including SO3/complexes, sulfuric acid-carbodiimide and chlorosulfonic acid-formamide [241]. It has been extensively reported that sulfated alginates exhibit anticoagulant activity and high affinity to bind HEP-binding growth factors [241,242]. This last hypothesis was also confirmed by Mhanna et al. [243], who synthesized alginates with variable degree of sulfation bearing sulfate groups at both hydroxyl free positions of uronic acid residues and investigated the effect of sulfation density on the affinity between the GAG-mimics and FGF-2. It was found that binding of FGF-2 was significantly greater for alginates with high sulfation degree compared to unmodified or low sulfated analogues [243].

## 5. Concluding Remarks

GAG, particularly ECM GAG, can be an asset in the development and design of new biomaterials given their biological and mechanical functions in native tissues. However, chemical modifications are often required for a successful stabilization and immobilization of GAG within hydrogel networks, due to their highly hydrophilic nature. As explored in this review, covalent crosslinking is the most widely used strategy to produce GAG-containing hydrogels, particularly the ones exclusively GAG-based. Nevertheless, reversible crosslinking, namely, dynamic covalent bonding and inclusion complexes can be used to develop systems with advanced features. Notably, functionalizations to improve biological performance rarely involve the incorporation of new bioactive cues, like cell-adhesive RGD peptides, in GAG backbone since GAG inherently possess such cell-interactive domains. Instead, biological performance of GAG is more often modulated and studied regarding sulfation degrees and patterns.

Hydrogels exclusively composed of GAG and their derivatives can be suitable platforms to deepen our understanding regarding their biological roles as well as to prospect their impact when incorporated in multi-component hydrogels. Indeed, the combination of GAG with other polymers allows a greater mimicry of the multicomponent and multifunctional profile of the native ECM since different sources and types of biomacromolecules with distinct biological roles can be included. In this case, bidirectional interactions can be promoted via chemical and physical bonding, enabling even dual crosslinking systems. In these hybrid systems, it is frequently required to improve GAG retention within the network to improve their biological performance, namely, their ability to recruit growth factors. Such approaches can be developed for producing carriers with higher growth factor loading/retention capacity, with modulated release, or to produce bioactive scaffolds able to recruit growth factors in situ. Besides their biological function, incorporation of GAG, particularly HA, can be primarily motivated by the improvement and modulation of rheological and mechanical properties of other systems.

However, it is noteworthy that crosslinking and GAG immobilization strategies affect not only the final mechanical properties, like stiffness or stress relaxation but also the biological function of immobilized GAG, as observed in reported studies combing GAG with gelatin or chitosan. This loss of biological function associated with modification or covalent crosslinking GAG or their derivatives can be partially circumvented by hybrid IPN hydrogels where GAG are preferably entrapped within a physical or chemical network formed by other materials.

Overall, HA is the most frequently used GAG in GAG-only hydrogels, mainly due to its biological role combined with adequate rheological and mechanical properties. In GAG-containing hybrid systems, apart from HA, most studies included CS or HEP, mainly motivated by their recognized biological roles and ability to interact with bioactive molecules.

Even though the added value of including GAG in hydrogels can be perceived by their biological functions, most reported studies still lack appropriate biological characterization. When hydrogel performance is evaluated regarding biological function, most studies are still poor, focusing mainly on single growth factor recruitment and cell viability, without deeply addressing cell activity/function. Proper controls are also often disregarded, with a lack of adequate comparison between GAG-containing hybrid networks and their GAG-free counterparts, in terms of biological and/or biomechanical performance. While this may blur the relevance of the reported benefits in formulations containing GAG, such comparisons would surely help to elucidate the clear roles of GAG in both the structural and biological features of such systems.

Finally, synthetically and naturally derived materials can be modified to mimic GAG and produce materials with higher control over purity, structure and sulfation degree/patterning. The vast majority of studies report HEP-mimicking polymers and are mainly characterized regarding their ability to interact with growth factors. Additionally, GAG-mimicking materials, of polysaccharidic nature and high sulfation degrees, may also be directly extracted from nature. In the field of GAG mimetics, special attention has been directed to the study of structure-activity relationship. Still, in the near future the development of glycomimetics should address the design of more complex structures, such as PG, in order to develop advanced materials with potential application in the fields of biomedicine and tissue engineering.
